# Procollagen 1 assembles into phase-separated condensates in the endoplasmic reticulum

**DOI:** 10.1083/jcb.202603129

**Published:** 2026-06-11

**Authors:** Soumya Bhattacharyya, Jose Wojnacki, Nathalie Brouwers, Vivek Malhotra

**Affiliations:** 1 Centre for Genomic Regulation (CRG), The Barcelona Institute for Science and Technology, Barcelona, Spain; 2 Universitat Pompeu Fabra (UPF), Barcelona, Spain; 3 ICREA, Barcelona, Spain

## Abstract

Procollagen I (PC1) is assembled into a trimer within the lumen of the endoplasmic reticulum (ER). In vitro, collagen trimers form rigid molecules reaching lengths of up to 400 nm, and this conformation is presumed to represent their assembled state in vivo. Here, we demonstrate that endogenous PC1 assembles into biomolecular condensates in the ER of activated human hepatic stellate cells. PC1 condensates form in response to increased collagen synthesis and are part of a multicomponent system enriched in the chaperones Hsp47 and calreticulin, as well as the disulfide isomerases PDIA1 and PDIA6, but notably lacking the unfolded protein sensor BiP. PC1 condensates localize to ER exit sites, a process mediated by TANGO1, and dissipate upon ER stress. We propose that this organization enables the accommodation of large quantities of PC1 in the ER lumen without triggering degradation. Furthermore, we suggest that PC1 within condensates is exported in a manner resembling liquid extrusion rather than as a rigid trimer.

## Teaser

In contrast to prevailing dogma, we report that procollagen 1 assembles into a liquid condensate within cells, resolving the long-standing problem of how cells accommodate and export high concentrations of these bulky molecules from the endoplasmic reticulum.

## Introduction

Procollagen I (PC1) is synthesized in the lumen of the endoplasmic reticulum (ER), where three α-chains (2 α1 and 1 α2) assemble into a triple-helical structure before being exported along the secretory pathway (Ishikawa, 2025; Malhotra and Erlmann, 2015; Makareeva and Leikin, 2007; Bruckner and Eikenberry, 1984; Beck et al., 1996). The export of PC1 from the ER depends on specialized ER exit sites (ERES) and requires the coordinated function of TANGO1, its binding partner cTAGE5, and the COPII coat machinery (Malhotra, 2024; Malhotra, 2025). In vitro biochemical and biophysical studies have shown that purified PC1 assembles into a rigid trimer that can reach lengths of up to ∼400 nm (Shoulders and Raines, 2009; Bella, 2016; Kotch and Raines, 2006). However, there is currently no direct experimental evidence demonstrating that PC1 adopts or is maintained in such a rigid, extended conformation within the ER lumen in vivo. Despite this, prevailing models assume that PC1 is secreted in an extended triple-helical form (Robinson et al., 2024; Bunel et al., 2024; Raote et al., 2023). This assumption presents a major conceptual problem, as canonical COPII vesicles are only ∼60–90 nm in diameter and therefore cannot accommodate a rigid cargo of several hundred nanometers in length.

TANGO1 has been proposed to resolve this size mismatch by functioning not as a classical cargo receptor, but as a scaffold that links ERES directly to ER–Golgi intermediate compartment (ERGIC) membranes (Raote et al., 2021). According to this model, TANGO1 organizes the fusion of ER and ERGIC membranes to generate a transient tunnel-like conduit, through which collagen is transferred directly from the ER lumen into post-ER compartments, bypassing the need for discrete, free COPII vesicles (Maeda et al., 2025; Yang et al., 2024; Liu et al., 2017; Raote et al., 2018; Raote et al., 2020; Saito et al., 2009; Saito et al., 2011). While this model elegantly addresses the problem of cargo size, it raises additional unresolved questions regarding the physical organization of PC1 within the ER lumen.

Specifically, if PC1 is assembled into a long, rigid trimer, it would be expected to reside largely parallel to the plane of the ER membrane due to spatial constraints. How such an extended molecule is selectively captured at ERES, engaged by TANGO1, and directed for export remains unclear.

Type I collagen is a major structural component of fibrotic tissue. In the liver, hepatic stellate cells (HSCs) become activated in response to injury and transforming growth factor-β (TGF-β) signaling, leading to increased synthesis and secretion of type I collagen (Kamm and McCommis, 2022; Mederacke et al., 2013). This raises a fundamental question: how does the ER accommodate and manage such high concentrations of PC1 under both physiological conditions and pathological states such as fibrosis?

## Results and discussion

### PC1 undergoes liquid–liquid phase separation

**Figure 1.**
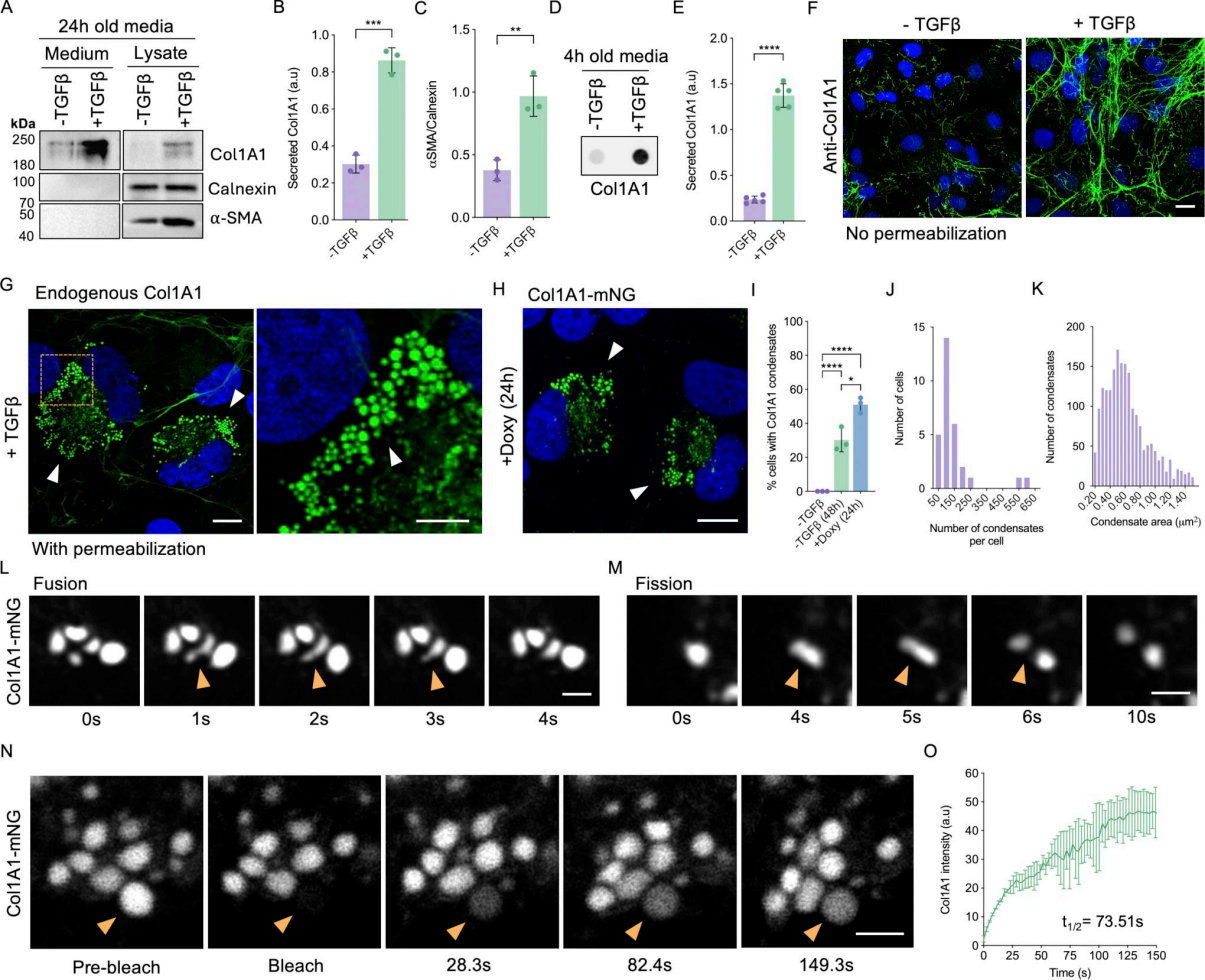
**PC1 assembles into biomolecular condensates. (A)** Western blot showing levels of secreted Col1A1 (medium) from 24-h-old media and intracellular expression levels of Col1A1, calnexin, and ⍺-SMA (lysate) in LX-2 cells grown with or without 10 ng/ml TGF-β for 48 h. **(B)** Expression levels of ⍺-SMA normalized to calnexin levels in cells grown in the absence or presence of TGF-β from three independent experiments (***P = 0.0048). **(C)** Levels of secreted Col1A1 normalized to intracellular calnexin levels from the 24-h-old culture medium from cells grown in the absence or presence of TGF-β from three independent experiments (**P = 0.0003). **(D)** Dot blot probed with anti-Col1A1 antibody showing levels of secreted Col1A1 from 4-h-old medium of cells grown with or without TGF-β. **(E)** Levels of secreted Col1A1 in 4-h-old culture medium of cells grown in the absence or presence of TGF-β from five independent experiments (****P < 0.0001). **(F)** Fluorescence images of LX-2 cells, fixed but not permeabilized and stained with anti-Col1A1 showing increased extracellular fibril formation due to increased secretion of Col1A1 upon TGF-β treatment (scale bar: 20 µm). **(G)** Fluorescence image of LX-2 cells grown with TGF-β (left), fixed and permeabilized, and stained with anti-Col1A1 showing the intracellular distribution of endogenous Col1A1 in distinct condensate-like structures (white arrowheads), and zoomed inset (right) highlighting their discrete spherical nature. Extracellular Col1A1 fibrils are also evident in the image since they are resistant to the permeabilization process (scale bars: 10 µm; inset: 5 µm). An expanded version of this image is presented in Fig. S1 D. **(H)** Fluorescence image of fixed and permeabilized LX-2 cells stably expressing Col1A1-mNG under a doxycycline-inducible promoter grown in the absence of TGF-β, 24 h after the addition of 1 µg/ml of doxycycline. White arrowheads show cells with distinct Col1A1 condensates (scale bar: 10 µm). **(I)** Percentage of LX-2 cells with Col1A1 condensates when grown without TGF-β, with TGF-β for 48 h or with doxycycline for 24 h but without TGF-β. A total of 229, 292, and 256 cells for the three conditions, respectively, from three independent experiments were analyzed (*P = 0.0124, ****P < 0.0001). **(J)** Histogram depicting the number of Col1A1 condensates per cell. 30 cells from three independent experiments were analyzed with condensate numbers ranging from 53 to 613 condensates per cell. **(K)** Histogram depicting condensate areas. A total of 1,934 condensates from 19 cells from three independent experiments were analyzed with a mode in the 0.50–0.55 µm^2^ range and a mean condensate area of 0.628 µm^2^. **(L)** Time-lapse images from Video 1 of LX-2 cells stably expressing Col1A1-mNG. The arrowhead indicates a fusion event between two condensates (scale bar: 2 µm). **(M)** Time-lapse images from Video 1 of LX-2 cells stably expressing Col1A1-mNG. The arrowhead indicates a fission event (scale bar: 2 µm). **(N)** Time-lapse images from Video 2 depicting FRAP of a Col1A1-mNG–expressing LX-2 cell. The arrowhead indicates condensate that was bleached and its subsequent recovery (scale bar: 2 µm). **(O)** Fluorescence recovery profile from two independent FRAP experiments. The data represent the mean and SD from the profiles. Source data are available for this figure: SourceData F1.

**Figure S1.**
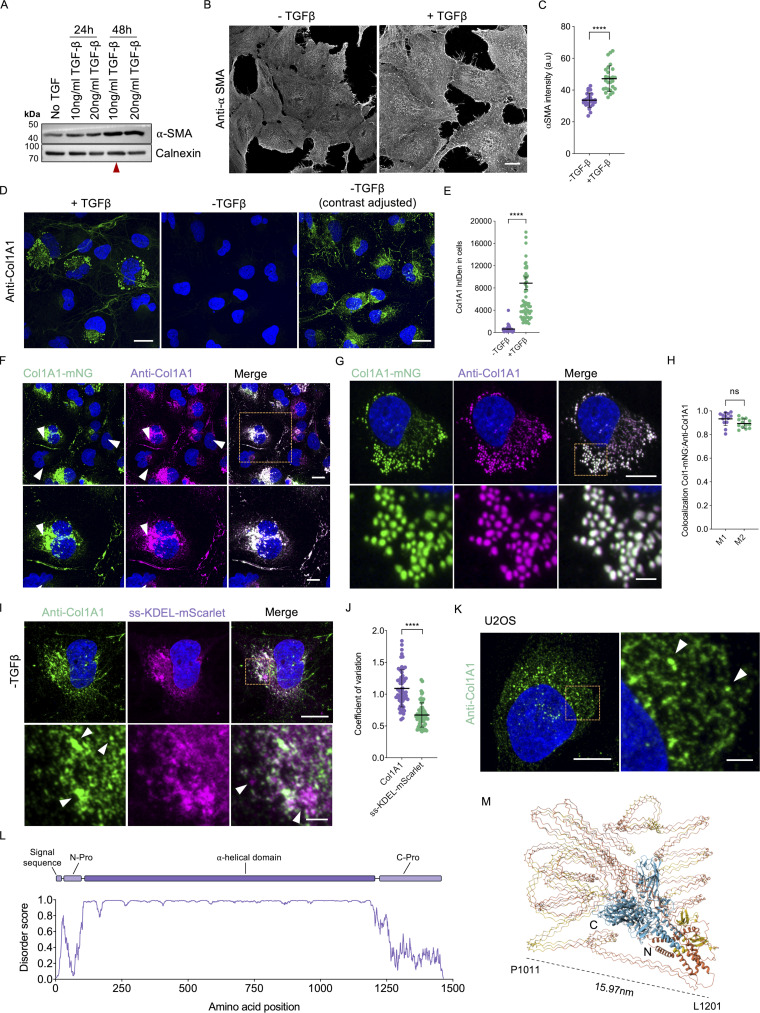
**PC1 is intrinsically predisposed for LLPS. (A)** Western blot showing an increase in ⍺-SMA levels in LX-2 cells upon treatment with 10 or 20 ng/ml of TGF-β for 24 and 48 h. Calnexin levels are used as loading controls. Throughout the results, +TGF-β refers to the condition indicated by the red arrowhead. **(B)** Fluorescence images of fixed LX-2 cells grown with or without TGF-β and stained using antibodies against ⍺-SMA (scale bar: 20 µm). **(C)** Plot quantifying mean ⍺-SMA intensity in cells grown with or without TGF-β. A total of 26 cells grown without TGF-β and 27 cells grown with TGF-β were analyzed (****P < 0.0001). **(D)** Fluorescence images of fixed LX-2 cells grown in the presence or absence of TGF-β. Images of cells grown without TGF-β with brightness and contrast equal to the +TGF-β condition (middle) and the same image with brightness and contrast enhanced (right) (scale bars: 20 µm). For comparison, an expanded version of Fig. 1 G is used as the +TGF-β image. **(E)** Plot quantifying fluorescence integrated density of intracellular COL1A1 in LX-2 cells grown with or without TGF-β. A total of 63 cells for each case from two independent experiments were analyzed (****P < 0.0001). **(F)** Fluorescence images of fixed LX-2 cells stably expressing Col1A1-mNG stained with antibodies against Col1A1 (top) and zoomed inset (bottom). Arrowheads indicate colocalization between Col1A1-mNG and anti-Col1A1 both inside the cell and in fibrils formed outside (scale bars: 10 µm; inset: 5 µm). **(G)** Fluorescence images of fixed LX-2 cells stably expressing Col1A1-mNG stained with antibodies against Col1A1 (top) and zoomed inset (bottom) showing colocalization between the two channels in Col1A1 condensates (scale bars: 10 µm; inset: 5 µm). **(H)** Manders’ colocalization coefficients M1 and M2 for images of LX-2 cells stably expressing Col1A1-mNG and stained with anti-Col1A1. A total of 13 ROIs from two independent experiments were analyzed (ns: not significant). **(I)** Fluorescence images of LX-2 cells grown in the absence of TGF-β, transfected with ss-KDEL-mScarlet, fixed, and stained for endogenous Col1A1 (top), and zoomed insets (bottom). White arrowheads in the Col1A1 channel indicate nonhomogeneous patches of Col1A1 and clusters resembling condensates, and those in the merged channel indicate patches of the ER that are not enriched in Col1A1 (scale bars: 10 µm; inset: 2 µm). **(J)** Plot comparing the CV in Col1A1 and ss-KDEL-mScarlet fluorescence across the same ROIs spanning the ER. A total of 59 ROIs from 20 cells from two independent experiments were analyzed (****P < 0.0001). **(K)** Fluorescence images of fixed U2OS cells stained for endogenous Col1A1 (left) and zoomed inset (right). White arrowheads indicate nonhomogeneous patches of Col1A1 and clusters resembling condensates (scale bars: 10 µm; inset: 2 µm). **(L)** Domain organization of Col1A1 and disorder scores across the protein predicted using IUPred2A (Erdős and Dosztányi, 2020). **(M)** Structure of a heterotrimer of PC1 with two ⍺1 chains and one ⍺2 chain predicted using the AlphaFold3 server (Abramson et al., 2024). The distance between residues P1011 and L1201 is indicated by the dashed line. ss-KDEL-mScarlet, signal-sequence-KDEL-mScarlet. Source data are available for this figure: SourceData FS1.

To investigate cellular responses to elevated PC1 synthesis, we used the immortalized human HSC line LX-2. It faithfully recapitulates features of activated stellate cells in vivo and has been extensively used to study signaling mechanisms regulating liver fibrosis, as well as to evaluate antifibrotic therapies and compounds (Xu et al., 2005; Yin et al., 2013; Shinn et al., 2024; Wang et al., 2023; Reiter et al., 2022; Carson et al., 2021; Castilho-Fernandes et al., 2011). Culturing cells with TGF-β resulted in an approximately threefold elevation in secreted type I collagen (Col1A1) in culture media after 24 h (Fig. 1, A–C). Because western blot detection of collagen is less reliable in media collected from shorter incubation periods, collagen secretion was additionally assessed using a dot blot–based assay. Media collected after only 4 h of incubation with activated cells showed an approximately sixfold increase in secreted Col1A1 relative to controls (Fig. 1, D and E). Activated HSCs exhibit increased levels of the contractility-promoting α-smooth muscle actin (α-SMA), and we further confirmed the activation of LX-2 cells by the threefold increase in α-SMA levels following TGF-β treatment (Fig. 1 C; and Fig. S1, A–C). As collagen fibrils remain associated with the cell surface, enhanced secretion was further verified by immunofluorescence staining of nonpermeabilized LX-2 cells. Cells cultured in the presence of TGF-β displayed markedly increased extracellular fibril formation, confirming that this system reliably induces elevated production and secretion of functional Col1A1 (Fig. 1 F).

Examination of intracellular collagen (PC1) organization in activated LX-2 cells revealed a striking nonuniform distribution. PC1 localized to discrete, highly intense spherical clusters, strongly resembling biomolecular condensates (Fig. 1 G, white arrows). These structures were most prominent in cells exhibiting the highest collagen signal, suggesting that their formation is linked to elevated collagen production (Fig. S1 D). Measuring PC1 fluorescence showed that activated LX-2 cells exhibited a ∼14-fold increase in intracellular type I collagen compared with untreated cells (Fig. S1 E). To determine whether elevated PC1 levels were sufficient to drive formation of these structures, we generated a LX-2 cell line stably expressing Col1A1–mNeonGreen (Col1A1-mNG) under a doxycycline-inducible promoter. Even in the absence of TGF-β, induction of collagen expression with doxycycline was sufficient to trigger formation of similar condensate-like structures (Fig. 1 H, white arrows). After 24 h of induction, ∼51% of cells displayed readily visible condensates, compared with ∼30% of cells treated with TGF-β for 48 h, indicating that condensate formation correlates with increased PC1 production (Fig. 1 I). The Col1A1-mNG construct was validated by immunostaining with an antibody against Col1A1, which showed strong colocalization between the fluorescent signal and endogenous collagen (Fig. S1, F–H). Moreover, extracellular fibrils containing Col1A1-mNG were detected, indicating that the tagged collagen was secreted and assembled into fibrillar structures similar to endogenous collagen (Fig. S1 F, white arrows). Quantitative analysis revealed that individual cells contained between 53 and 613 distinct PC1 condensates (Fig. 1 J). Condensate size varied considerably, with a modal surface area of 0.50–0.55 μm^2^ and a mean area of 0.628 μm^2^ (Fig. 1 K), and diameters ranging between 0.5 and 1.4 μm. Live-cell imaging of cells expressing Col1A1-mNG revealed that these structures were dynamic, exhibiting frequent fusion and fission events characteristic of liquid-like assemblies (Video 1; and Fig. 1, L and M). Consistent with this behavior, fluorescence recovery after photobleaching (FRAP) experiments demonstrated rapid exchange of Col1A1 molecules within condensates at timescales typical of previously described cellular biomolecular condensates (Leder et al., 2025; Li et al., 2022; Muzzopappa et al., 2022) (Video 2; and Fig. 1, N and O).

**Video 1.** **PC1 condensates undergo fission and fusion.** Time-lapse confocal microscopy showing PC1 condensates undergoing fusion and fission in a cell, corresponding to Fig. 1, L and M. The blue arrow indicates a fusion event followed by fission into two separate condensates. The red arrows indicate fusion events. Fluorescence signal in gray is from Col1A1-mNG. Frames were acquired at a time interval of 1 s, and the movie is played at 8 fps (scale bar: 5 μm).

**Video 2.** **PC1 fluorescence within condensates recovers after photobleaching.** Time-lapse confocal microscopy showing fluorescence recovery of PC1 in a condensate after photobleaching (FRAP) indicated by the red arrow, corresponding to Fig. 1 N. Fluorescence signal in gray is from Col1A1-mNG. Frames were acquired at a time interval of 2.5 s, and the movie is played at 10 fps (scale bar: 2 μm).

Because distinct condensates were apparent in response to elevated PC1 synthesis, we next examined collagen distribution under conditions of lower expression levels. Even when grown without TGF-β, PC1 distribution in LX-2 cells was heterogeneous and included smaller patches and clusters (Fig. S1 I, white arrows in the Col1A1 channel). To confirm this heterogeneous distribution did not arise from projections of the ER geometry, we transfected LX-2 cells with a construct encoding a signal sequence and the ER retention sequence KDEL tagged to mScarlet (ss-KDEL-mScarlet) and stained for endogenous PC1. While the ss-KDEL-mScarlet fluorescence was diffuse and resembled the entirety of the ER, the PC1 signal was in discrete clusters, which remained segregated from the ss-KDEL-mScarlet signal in multiple regions (Fig. S1 I, white arrows in the merged channel). Quantitation of the coefficient of variation (CV) between PC1 and ss-KDEL-mScarlet fluorescence in the ER revealed a significantly higher CV for PC1 indicating PC1 clusters occupy distinct zones in the ER (Fig. S1 J). Furthermore, live-cell imaging of cells with lower expression levels revealed numerous PC1 patches that underwent cycles of fusion and fission, indicating that the liquid-like behavior persists even when large spherical condensates are not evident (Video 3). To determine whether this behavior was specific to HSCs, we examined PC1 distribution in the human osteosarcoma cell line U2OS. Endogenous PC1 displayed a similarly heterogeneous distribution with frequent clustering, suggesting that this liquid-like behavior is a general property of PC1 across multiple cell types (Fig. S1 K, white arrows).

**Video 3.** **Liquid-like nature of PC1 in the ER.** Time-lapse confocal microscopy of Col1A1-mNG in a cell with low levels of Col1A1 expression showing the dynamics of clustered patches of PC1 in the ER (left) and zoomed inset of the boxed region (right). The white arrow indicates frequent fusion and fission events by the smaller PC1 clusters. Col1A1-mNG is colored green and Hoechst staining in blue. Frames were acquired at a time interval of 1 s, and the movie is played at 10 fps (scale bar: 5 μm; inset: 2 μm).

A major driver for liquid–liquid phase separation (LLPS) is a high degree of molecular disorder (Shin and Brangwynne, 2017; Alberti et al., 2019; Wang et al., 2018). Analysis of the amino acid composition of Col1A1 indicated a high disorder propensity within its α-helical domain (Fig. S1 L). Glycine and proline comprise 26.7% and 19% of the total residues, respectively, with frequent P–G repeats distributed throughout this region, features likely to contribute to its disordered character. Additionally, structural prediction of the PC1 trimer also reveals a largely disordered conformation for the α-helical domain (Fig. S1 M). Together, these observations suggest that PC1 is intrinsically predisposed for LLPS. However, at present, we cannot conclude that the fully assembled trimer is disordered and whether this contributes directly to PC1 condensate formation. Further investigation is necessary to understand the folded state of PC1 within the condensates.

### PC1 condensates are enriched in chaperones and dissipate upon ER stress

**Figure 2.**
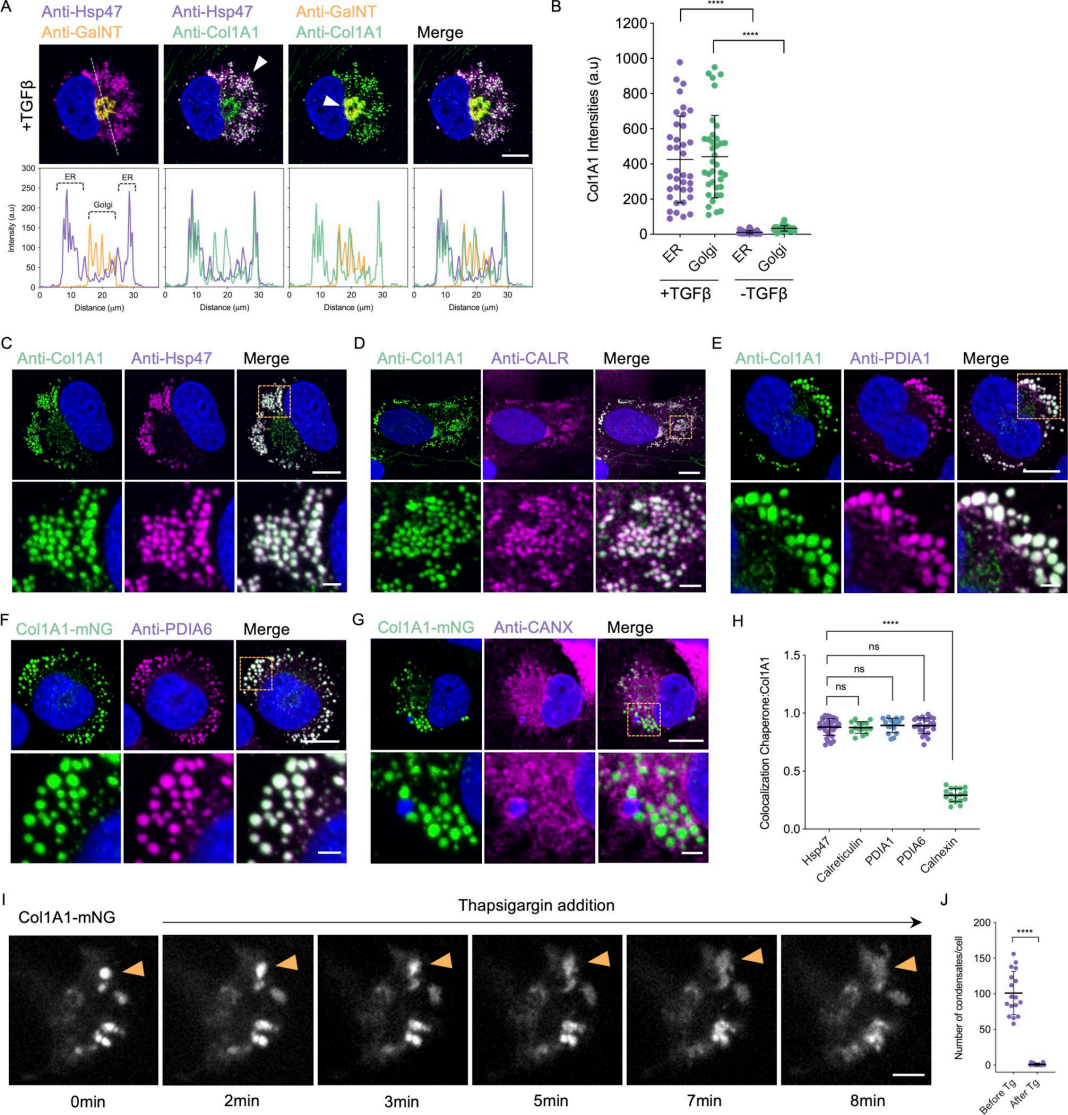
**PC1 condensates are enriched in chaperones and sensitive to ER stress. (A)** Fluorescence images of fixed LX-2 cells activated with TGF-β and stained for antibodies against endogenous Col1A1 (green), Hsp47 (magenta), and GalNT (orange) (top), and fluorescence intensity profiles (bottom) along the dashed white line. White arrows show distinct Col1A1 pools in the ER and Golgi, demonstrating PC1 condensates are localized in the ER (scale bar: 10 µm). **(B)** Plot quantifying Col1A1 intensities in the ER and Golgi in LX-2 cells grown with or without TGF-β, showing increased collagen levels in both organelles upon TGF-β treatment. A total of 38 different cells with TGF-β and 34 cells without TGF-β from two independent experiments were analyzed (****P < 0.0001). **(C)** Fluorescence images of fixed LX-2 cells activated with TGF-β and stained for antibodies against endogenous Col1A1 (green) and Hsp47 (magenta) (top), and zoomed insets (bottom) (scale bar: 10 µm; inset: 2 µm). **(D)** Fluorescence images of fixed LX-2 cells activated with TGF-β and stained for antibodies against endogenous Col1A1 (green) and calreticulin (CALR) (magenta) (top), and zoomed insets (bottom) (scale bar: 10 µm; inset: 2 µm). **(E)** Fluorescence images of fixed LX-2 cells activated with TGF-β and stained for antibodies against endogenous Col1A1 (green) and protein disulfide isomerase A1 (PDIA1) (magenta) (top), and zoomed insets (bottom) (scale bar: 10 µm; inset: 2 µm). **(F)** Fluorescence images of fixed LX-2 cells stably expressing Col1A1-mNG (green) and protein disulfide isomerase A6 (PDIA6) (magenta) (top), and zoomed insets (bottom) (scale bar: 10 µm; inset: 2 µm). **(G)** Fluorescence images of fixed LX-2 cells stably expressing Col1A1-mNG (green) and calnexin (CANX) (magenta) (top), and zoomed insets (bottom) (scale bar: 10 µm; inset: 2 µm). **(H)** Manders’ colocalization coefficient M2 for Col1A1 and the respective chaperones. A total of 33, 15, 16, 24, and 17 cells for Hsp47, CALR, PDIA1, PDIA6, and CANX from two independent experiments were analyzed (ns—not significant; ****P < 0.0001). **(I)** Time-lapse images from Video 4 showing dissipation of PC1 condensates upon thapsigargin treatment (scale bar: 2 µm). **(J)** Plot quantifying PC1 condensate number per cell before and after thapsigargin treatment. A total 17 cells from two independent experiments were analyzed (****P < 0.0001).

We visualized PC1 localization relative to established markers of the ER and Golgi. Immunofluorescence analysis using the ER luminal chaperone heat shock protein 47 (Hsp47) and the Golgi enzyme polypeptide N-acetylgalactosaminyltransferase (GalNT) revealed two spatially distinct pools of PC1 (Fig. 2 A, green trace w.r.t. magenta and orange trace, white arrows). One population colocalized with GalNT in the Golgi, whereas a separate pool colocalized with Hsp47 in the ER. Notably, PC1 condensates observed in activated HSCs colocalized strongly with Hsp47, indicating that these condensates form within the ER lumen (Fig. 2 A). In cells treated with TGF-β where distinct condensates were apparent, PC1 levels increased in both the ER and Golgi compared with untreated cells, consistent with elevated collagen secretion during HSC activation (Fig. 2 B). The increased abundance of PC1 was accompanied by enhanced condensate formation, suggesting that condensate assembly is concentration-dependent.

Hsp47 is a well-characterized ER chaperone that selectively recognizes properly folded collagen triple helices and is required for collagen stability (Nagata et al., 1986; Satoh et al., 1996; Widmer et al., 2012; Makareeva and Leikin, 2007). Depletion of Hsp47 results in PC1 misfolding and aggregation (Kawasaki et al., 2015; Syx et al., 2021; Widmer et al., 2012). The strong enrichment of Hsp47 within PC1 condensates therefore suggests that collagen present in these structures is correctly assembled into triple-helical trimers (Fig. 2 C). We next examined the localization of additional ER chaperones involved in collagen folding, including calreticulin and protein disulfide isomerase PDIA1 (Van Duyn Graham et al., 2010; Wilson et al., 1998). Both proteins were enriched within PC1 condensates, suggesting that these structures concentrate multiple collagen-folding and processing factors (Fig. 2, D and E). Recent studies have described multichaperone condensates in the ER that facilitate protein folding, with their assembly driven by the calcium-dependent oligomerization of PDIA6 (Leder et al., 2025; Lee et al., 2025). Although PDIA6 has not previously been implicated in collagen folding, we tested whether it is present within PC1 condensates. Immunofluorescence analysis revealed that PDIA6 is also enriched in condensates suggesting a role in PC1 processing (Fig. 2 F). In contrast, the ER transmembrane chaperone calnexin was not enriched in these structures (Fig. 2, G and H). Together, these observations indicate that PC1 assembles into a multichaperone condensate within the ER lumen. PDIA6 condensates disperse upon triggering ER stress (Leder et al., 2025). To test whether PC1 condensates behave the same, we treated cells with thapsigargin to deplete ER calcium stores, thus triggering ER stress. Upon thapsigargin treatment, PC1 condensates rapidly lost their spherical morphology and dispersed within minutes (Video 4; and Fig. 2, I and J). These findings demonstrate that the assembly of PC1 condensates is calcium-dependent and their integrity is lost upon triggering ER stress.

**Video 4.** **PC1 condensates dissipate upon thapsigargin addition.** Time-lapse confocal microscopy showing dissipation of PC1 condensates upon addition of 1 μM thapsigargin as indicated by the white arrow, corresponding to Fig. 2 I. Col1A1-mNG is colored in green. Frames were acquired at a time interval of 60 s, and the movie is played at 8 fps (scale bar: 5 μm).

### PC1 condensates are not fated for degradation and do not trigger cellular stress responses

**Figure 3.**
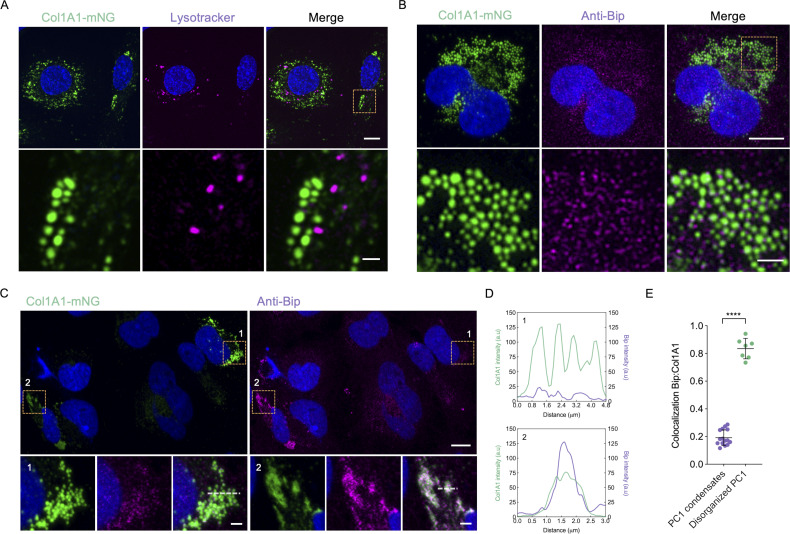
**PC1 in condensates is not targeted for degradation. (A)** Fluorescence images of LX-2 cells stably expressing Col1A1-mNG (green) stained with LysoTracker Red (magenta) (top), and zoomed inset (bottom) showing that lysosomes are not in the vicinity of PC1 condensates. Images are stills corresponding to Video 5 (scale bar: 10 µm; inset: 2 µm). **(B)** Fluorescence images of fixed LX-2 cells stably expressing Col1A1-mNG (green) and stained using antibodies against the chaperone Bip (magenta) (top), and zoomed inset (bottom) showing Bip is excluded from PC1 condensates (scale bar: 10 µm; inset: 2 µm). **(C)** Fluorescence images of fixed LX-2 cells stably expressing Col1A1-mNG (green) and stained using antibodies against Bip (magenta) showing a cell with PC1 condensates (1) and another with disorganized PC1 (2) (top), and zoomed insets of regions corresponding to 1 and 2 (bottom) (scale bars: 10 µm; inset: 2 µm). **(D)** Fluorescence intensity profiles corresponding to the dashed white lines in zoomed insets 1 and 2 of D for Col1A1-mNG (green) and anti-Bip (magenta) in PC1 condensates and disorganized PC1 highlighting increased Bip accumulation in disorganized PC1 patches. **(E)** Manders’ colocalization coefficient M2 for Col1A1-mNG and anti-Bip in cells with either PC1 condensates or disorganized PC1. A total of 15 cells with PC1 condensates and seven cells with disorganized PC1 from two independent experiments were analyzed (****P < 0.0001).

**Figure S2.**
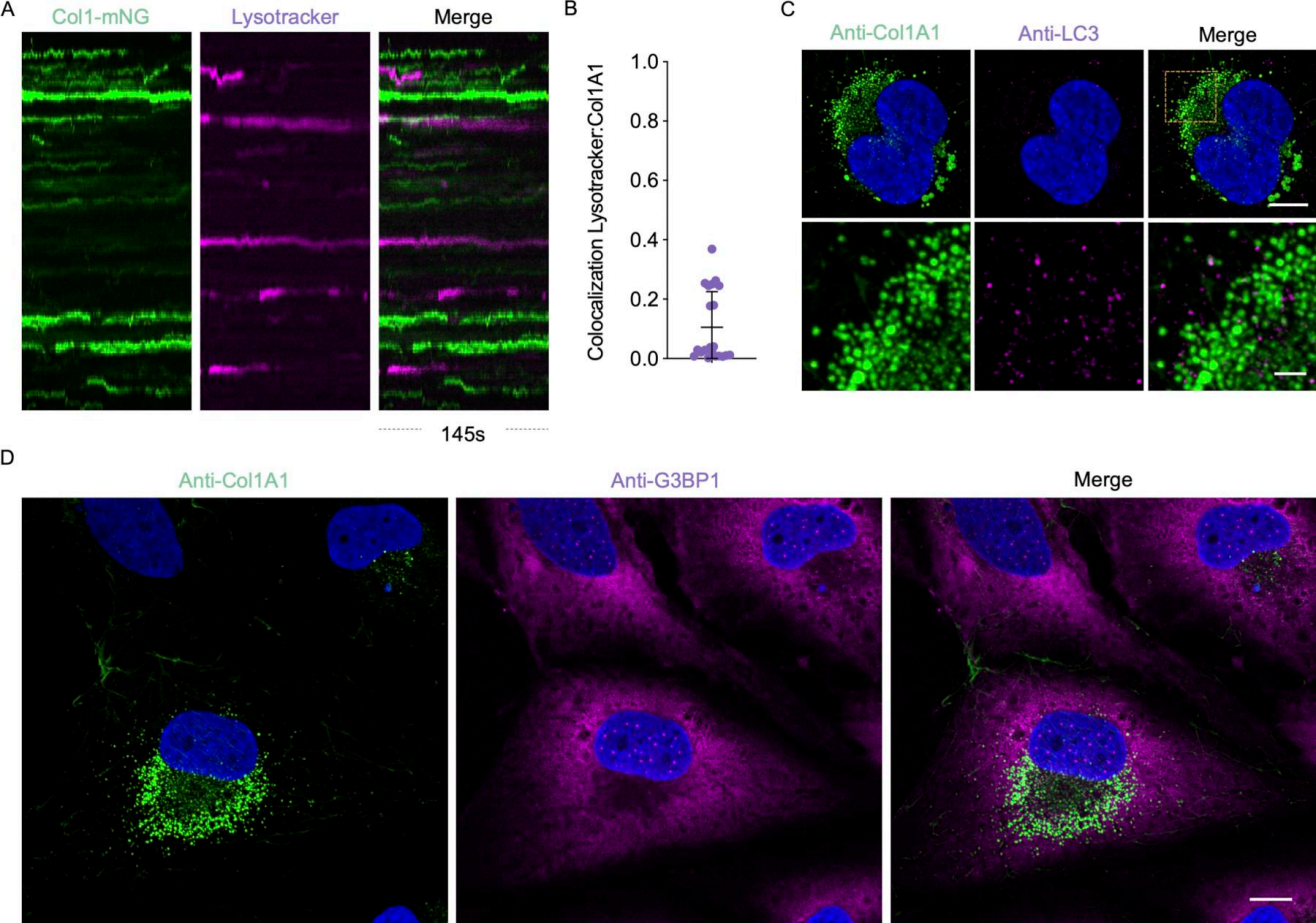
**PC1 condensates do not trigger a cellular stress response. (A)** Kymograph corresponding to a line drawn through a cell in Video 5 showing no overlap between the Col1-mNG (green) and LysoTracker (magenta) traces. **(B)** Manders’ colocalization coefficient M2 for Col1A1-mNG and LysoTracker Red in 21 cells from two independent experiments showing low colocalization between PC1 condensates and lysosomes. **(C)** Fluorescence images of fixed activated LX-2 cells stained with antibodies against endogenous Col1A1 (green) and LC3 (magenta) (top), and zoomed insets (bottom) showing PC1 condensates are not in the vicinity of autophagosomes (scale bars: 10 µm; inset: 2 µm). **(D)** Fluorescence images of fixed activated LX-2 cells stained with antibodies against endogenous Col1A1 (green) and G3BP1 (magenta) showing homogeneous G3BP1 signal indicating cells with PC1 condensates are not eliciting an overall stress response (scale bar: 10 µm).

PC1 degradation occurs through a noncanonical microautophagy-like process whereby lysosomes engulf collagen accumulated at LC3-marked ERES (Omari et al., 2018; Makareeva et al., 2025). To determine whether PC1 in condensates are *en route* for degradation, we performed live-cell imaging using LysoTracker to label lysosomes. These experiments revealed that PC1 condensates do not localize in proximity to lysosomes, nor colocalize with LC3-positive autophagosomes, arguing against their identity as cargo destined for degradation (Video 5, Fig. 3 A, and Fig. S2, A–C). We next asked whether the presence of condensates elicited a cellular stress response. Stress granule formation was assessed using Ras GTPase-activating protein-binding protein 1 (G3BP1), a key mediator of stress granule assembly (Yang et al., 2020), which remained diffusely distributed (Fig. S2 D). G3BP1 did not form clusters, indicating that PC1 condensates are not associated with stress granules and that their formation does not trigger a cellular stress response. To further test whether condensates arise from protein misfolding, we examined the localization of the ER chaperone BiP. BiP recognizes exposed hydrophobic regions on misfolded proteins and dissociates from IRE1, causing IRE1 oligomerization and activation of the unfolded protein response (UPR) (Gardner et al., 2013; Pincus et al., 2010; Kimata et al., 2003). In fibroblasts from patients with osteogenesis imperfecta, Bip binds PC1 with mutations at its C terminus (Chessler and Byers, 1993) and has also been shown to bind aggregated misfolded collagen (John et al., 1993). In activated HSCs with PC1 condensates, BiP was not enriched within the condensates and instead appeared as small punctate structures in their vicinity (Fig. 3 B). In contrast, cells displaying irregular and disorganized PC1 clusters showed strong BiP association (Fig. 3, C–E, magenta and green traces in 1 w.r.t. 2). This distinction allowed us to differentiate between PC1 that is correctly folded in condensates from either nascent PC1 chains or likely misfolded pools of PC1. Together, our results indicate that cells assemble multichaperone condensates to accommodate large quantities of PC1 and prevent it from being degraded by the UPR machinery.

**Video 5.** **Lysosomes do not colocalize with PC1 condensates.** Time-lapse confocal microscopy of cells with PC1 condensates (green) and stained with LysoTracker Red (magenta) showing condensates are not in the vicinity of lysosomes, corresponding to Fig. 3 A and Fig. S2 A. Nuclear staining with Hoechst colored in blue. Frames were acquired at a time interval of 1 s, and the movie is played at 10 fps (scale bar: 10 μm).

### TANGO1 captures PC1 condensates at ERES

**Figure 4.**
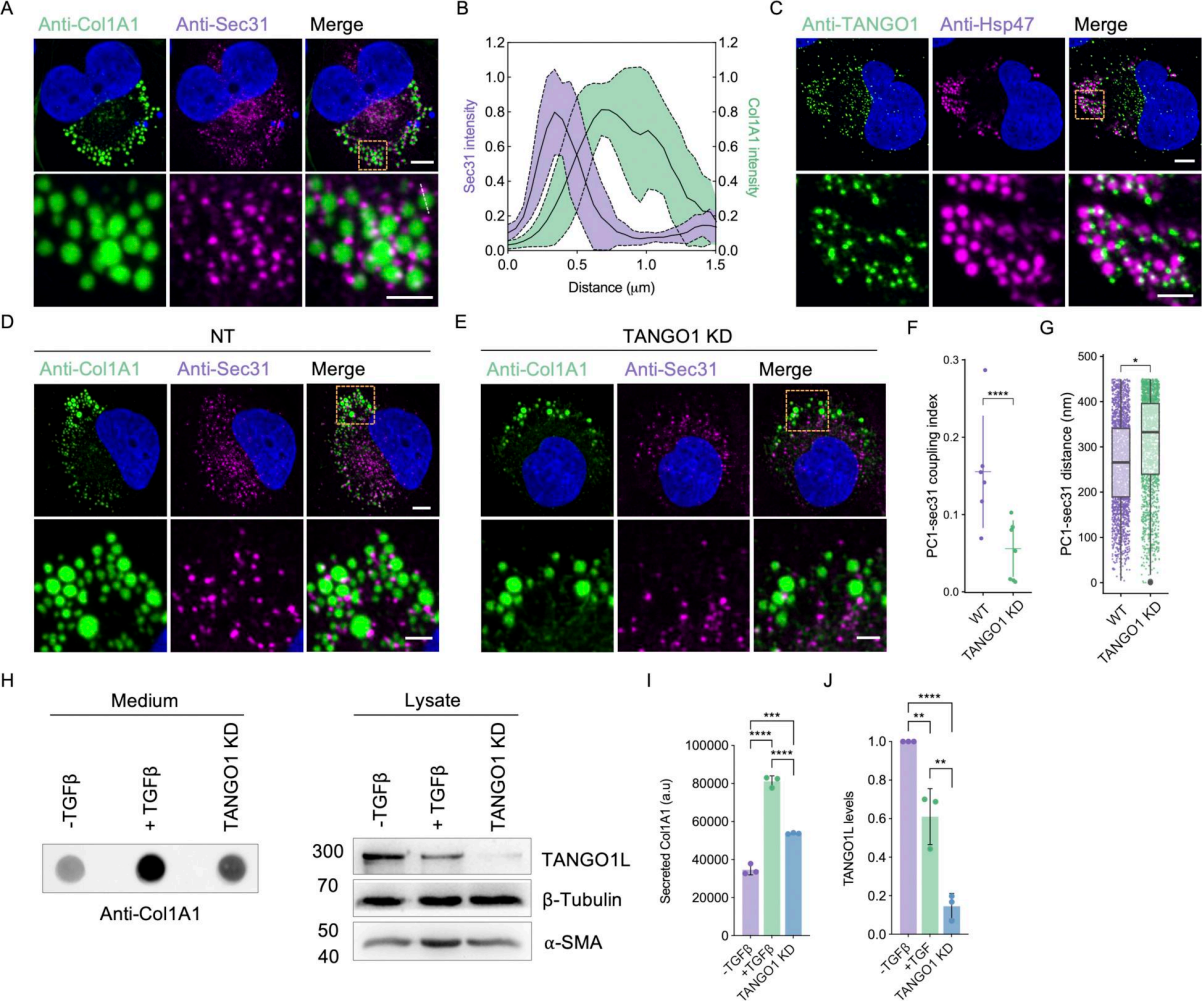
**PC1 condensates are captured by TANGO1 at ERES. (A)** Fluorescence images of fixed LX-2 cells activated with TGF-β and stained for antibodies against endogenous Col1A1 (green) and Sec31 (magenta) (top), and zoomed insets (bottom) (scale bars: 5 µm; inset: 2 µm). **(B)** Fluorescence intensity profiles corresponding to lines similar to the dashed white line in the zoomed inset in A for anti-Col1A1 (green) and anti-Sec31 (magenta) showing the proximity of ERES to PC1 condensates. The black lines represent the mean, and the shaded region within the dotted lines represents the SD of intensity profiles from 14 different PC1 condensates. **(C)** Fluorescence images of fixed LX-2 cells activated with TGF-β and stained for antibodies against endogenous TANGO1 (green) and Hsp47 (magenta) (top), and zoomed insets (bottom) (scale bars: 5 µm; inset: 2 µm). **(D)** Fluorescence images of fixed LX-2 cells activated with TGF-β and treated with NT siRNAs and stained using antibodies against endogenous Col1A1 (green) and Sec31 (magenta) (top), and zoomed insets (bottom) (scale bars: 5 µm; inset: 2 µm). **(E)** Fluorescence images of fixed LX-2 cells activated with TGF-β and treated with siRNAs against TANGO1 (TANGO1 KD) and stained using antibodies against endogenous Col1A1 (green) and Sec31 (magenta) (top), and zoomed insets (bottom) (scale bars: 5 µm; inset: 2 µm). **(F)** Plot showing decreased PCI condensate–Sec31 coupling upon TANGO1 KD. Data show values representing the mean and SD from six cells in NT and eight cells in TANGO1 KD from two independent experiments (****P < 0.0001). **(G)** Plot showing increased distances between PC1 condensates and Sec31 puncta upon TANGO1 KD. A total of 2,407 and 1,940 condensates were analyzed from 6 cells in NT and eight cells in TANGO1 KD, respectively, from two independent experiments (*P = 0.0193). **(H)** Dot blot probed with anti-Col1A1 antibody showing levels of secreted Col1A1 from 4-h-old medium of cells (medium) grown in the absence of TGF-β and with TGF-β, and treated with NT siRNAs or grown with TGF-β and treated with siRNAs against TANGO1 (TANGO1 KD), and western blot showing intracellular levels (lysate) of TANGO1, β-tubulin, and ⍺-SMA in cells grown in the same conditions corresponding to cells used for the dot blot. **(I)** Levels of secreted Col1A1 in 4-h-old culture medium of cells grown under conditions specified in H (***P = 0.0003, ****P < 0.0001, ****P < 0.0001; top to bottom). **(J)** Intracellular levels of TANGO1 in cells grown under conditions specified in H (****P < 0.0001, **P = 0.0097, **P = 0.0073; top to bottom). NT, nontargeting. Source data are available for this figure: SourceData F4.

**Figure S3.**
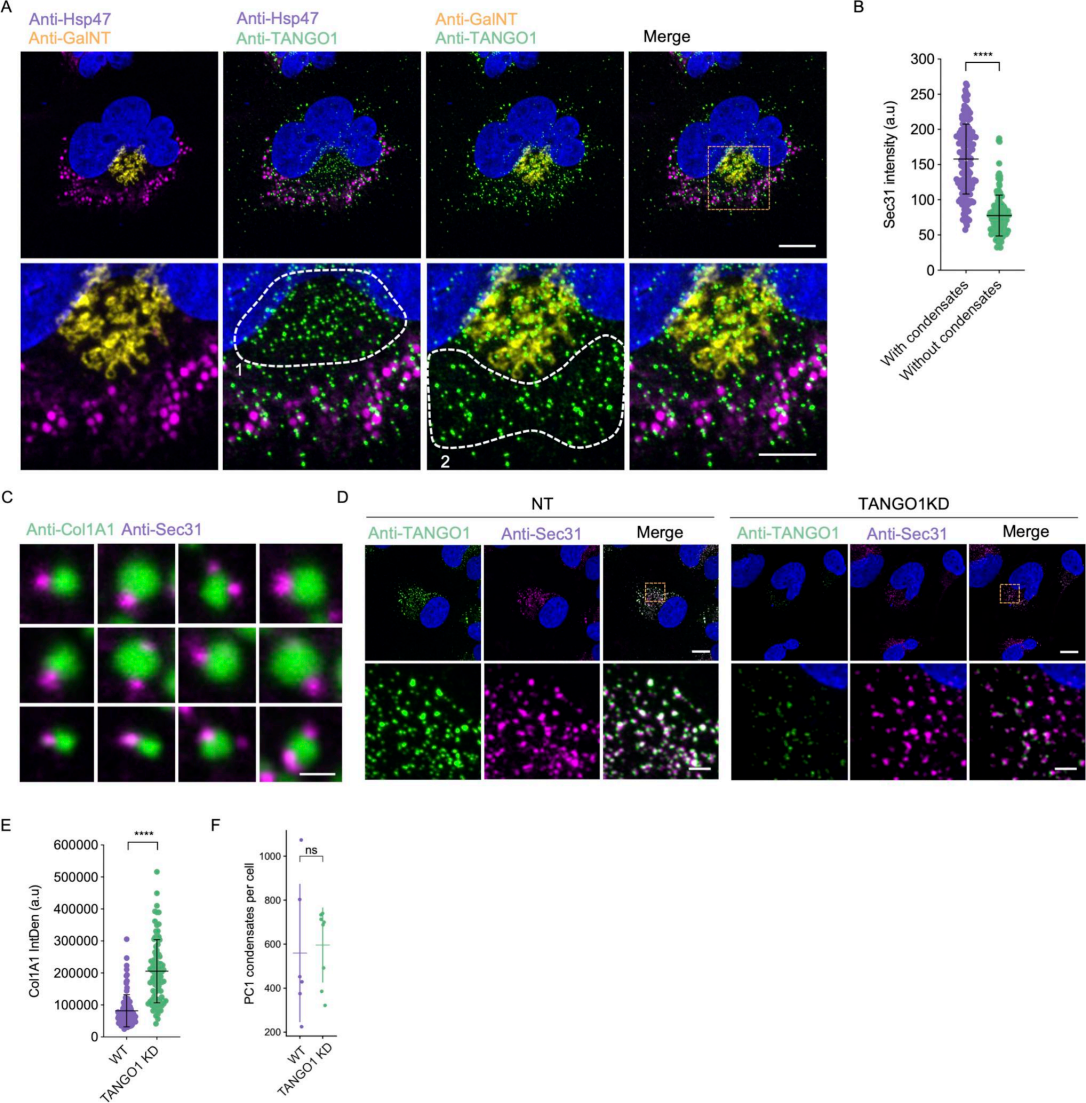
**Functional division of labor among ERES. (A)** Fluorescence images of activated LX-2 cells stained for endogenous Hsp47 (magenta), GalNT (yellow), and TANGO1 (green) (top), and zoomed insets (bottom) showing two distinct pools of ERES bounded by dashed white lines. Region 1 shows the ERES pool associated with the Golgi, and region 2 shows the ERES pool associated with Col1A1 condensates (scale bars: 10 µm; inset: 5 µm). **(B)** Plot comparing the fluorescence intensity of Sec31 puncta in ERES that are associated with PC1 condensates vs those that are not associated with PC1 condensates. A total of 144 ERES associated with condensates and 127 ERES not associated with condensates from two independent experiments were analyzed (****P < 0.0001). **(C)** Panel showing different Col1A1 condensates (green) and associated Sec31 puncta (magenta) used to generate the fluorescence intensity profiles in Fig. 4 B (scale bar: 1 µm). **(D)** Fluorescence images of activated LX-2 cells treated with either NT siRNAs or siRNAs targeting TANGO1 (TANGO1 KD) stained for antibodies against endogenous TANGO1 (green) and Sec31 (magenta) (top), and zoomed insets (bottom) showing reduced TANGO1 levels upon knockdown (scale bars: 10 µm; inset: 5 µm). **(E)** Plot comparing Col1A1 integrated density in cells treated with either NT siRNAs (WT) or siRNAs targeting TANGO1 (TANGO1 KD). 97 cells in WT and 88 cells in TANGO1 KD from two independent experiments were analyzed (****P < 0.0001). **(F)** Plot quantifying the number of PC1 condensates in cells treated with either NT siRNAs (WT) or siRNAs targeting TANGO1 (TANGO1 KD) (ns: not significant). NT, nontargeting.

The absence of BiP from PC1 condensates, together with their enrichment in chaperones such as Hsp47, suggested that PC1 within these structures are ready to be exported from the ERES. ERES are molecular assemblies distributed throughout the ER that mark sites of cargo export (Farhan et al., 2025), and we next asked whether PC1 condensates are associated with ERES. To address this, we stained cells for the COPII coat subunit Sec31. ERES were positioned immediately adjacent to PC1 condensates, with one or more Sec31-positive puncta frequently observed at the condensate periphery (Fig. 4, A and B; and Fig. S3 C). We further analyzed the localization of TANGO1, which, to our knowledge, is the only resident ERES protein that spans the ER membrane and extends into the ER lumen. Costaining for TANGO1 and Hsp47 showed that TANGO1, like Sec31, was enriched at the periphery of condensates (Fig. 4 C). Notably, we could distinguish two spatially distinct pools of ERES: a perinuclear population associated with the Golgi region and a more peripheral population associated with PC1 condensates (Fig. S3 A, regions 1 and 2, white dashed lines). In accordance with previous work showing ERES challenged with increased cargo load are bigger (Farhan et al., 2008), quantitation of Sec31 fluorescence revealed that the intensity of ERES puncta associated with PC1 condensates is significantly higher than those that are not (Fig. S3 B). These observations are also consistent with recent work from our laboratory demonstrating the existence of at least two classes of ERES, and suggest a functional division of labor among exit sites within the same cell (Saxena et al., 2024).

Because Hsp47 binds the SH3-like domain of TANGO1 and thereby provides a molecular link between TANGO1 and PC1 (Ishikawa et al., 2016), we next tested whether TANGO1 contributes to the assembly or positioning of PC1 condensates at ERES. We used siRNAs directed against the luminal domain of TANGO1 to selectively deplete the long isoform of TANGO1. PC1 condensates were still formed after TANGO1 knockdown, indicating that TANGO1 is not required for condensate formation per se. However, TANGO1 depletion caused a marked reduction in the association of PC1 condensates with ERES, as quantified by the coupling index between PC1 condensates and Sec31 puncta (Fig. 4, D–F). Consistent with this, the average distance between PC1 condensates and Sec31-positive structures increased in TANGO1-depleted cells relative to controls (Fig. 4 G). Importantly, loss of TANGO1 also reduced secretion of Col1A1 by ∼1.5-fold compared with control cells and resulted in increased intracellular retention of Col1A1, indicating that capture of PC1 condensates at ERES is important for efficient secretion (Fig. 4, H and I; and Fig. S3 E). Efficient depletion of TANGO1 was confirmed by both immunofluorescence and immunoblotting (Fig. 4 J and Fig. S3 D). Interestingly, TANGO1 depletion did not significantly alter the number of distinctly spherical condensates per cell (Fig. S3 F), but the increased Col1A1 retention resulted in a more irregular, disorganized distribution of collagen in the ER and these differences in organization could regulate how collagen is segregated for subsequent degradation, in line with our observations on Bip accessibility to condensates. Together, these data suggest that PC1-containing condensates are primed for secretion and identify TANGO1 as a key factor in their capture at ERES.

In vitro studies have long established that collagen trimers are extended, rigid molecules that can reach lengths of up to 400 nm. This structural paradigm presents a fundamental topological challenge: the ER lumen, with a thickness of only 30–60 nm, appears insufficient to accommodate or export such elongated assemblies in a perpendicular orientation. How these large cargoes are packaged and conveyed through the early secretory pathway has therefore remained an unresolved question.

Our findings offer a conceptual resolution to this paradox. Rather than existing as rigid, rodlike trimers within the ER lumen, PC1 adopts a dynamic, liquid-like condensate state that is sufficiently pliable to conform to the shape and volume of the ER lumen. These condensates are enriched in the collagen-specific chaperone Hsp47, which selectively binds folded trimeric PC1, as well as calreticulin and the disulfide isomerases PDIA1 and PDIA6. Notably, they exclude the transmembrane lectin chaperone calnexin and the unfolded protein sensor BiP, indicating that PC1 within these assemblies has progressed beyond early folding checkpoints and is not targeted for degradation.

While the ER has recently emerged as a site for the formation of biomolecular condensates (Leder et al., 2025; Lee et al., 2025), PC1 condensates are functionally and compositionally distinct from previously described assemblies. In contrast to proinsulin-containing condensates, which exclude calreticulin and retain BiP, PC1 condensates are enriched in calreticulin and devoid of BiP, consistent with a more advanced stage of protein maturation. Upon TGF-β–induced upregulation of collagen synthesis, both the number and size of PC1 condensates increase dramatically, with structures reaching up to a micron in diameter. Importantly, this expansion does not elicit ER stress or degradation pathways, suggesting that condensate formation provides a protective environment that accommodates high biosynthetic load while preserving proteostasis. Furthermore, the close spatial association of PC1 condensates with ERES underscores their direct engagement with the secretory machinery. Together, these observations support a model in which the ER lumen is not a passive conduit but an adaptive environment capable of organizing distinct condensate populations tailored to specific cargoes.

An intriguing parallel emerges when considering the mechanism of cargo capture at ERES in relation to the nuclear pore complex. In the nucleus, ribonucleoprotein particles are selectively engaged by phenylalanine–glycine repeat domains within nucleoporins, facilitating their translocation (Schmidt and Görlich, 2016). Similarly, the tyrosine and glycine-rich domain in PEX13 facilitates peroxisomal import (Skowyra et al., 2024). Analogously, the proline- and glycine-rich regions of PC1 may be recognized by intrinsically disordered domains of TANGO1 at ERES. This conceptual similarity suggests that both systems exploit multivalent, low-affinity interactions to concentrate and transfer large macromolecular assemblies, pointing to a potentially conserved physical principle underlying selective export across distinct cellular compartments.

These insights prompt a reassessment of the physical form in which PC1 exits the ER. TANGO1, together with COPII components, orchestrates the formation of a specialized export conduit. Within the ER lumen, TANGO1 engages Hsp47-bound PC1, while its cytoplasmic domains interface with Sec23 and additional factors including Sec16, cTAGE5, and the NRZ complex, thereby recruiting ERGIC membranes (Raote et al., 2018; Raote et al., 2021). Fusion of these membranes establishes a transient channel connecting the ER to downstream compartments. Previous models have envisaged the passage of rigid PC1 trimers through this conduit (Bunel et al., 2024). However, our data support an alternative mechanism in which PC1 is transported in a liquid-like state.

**Figure 5.**
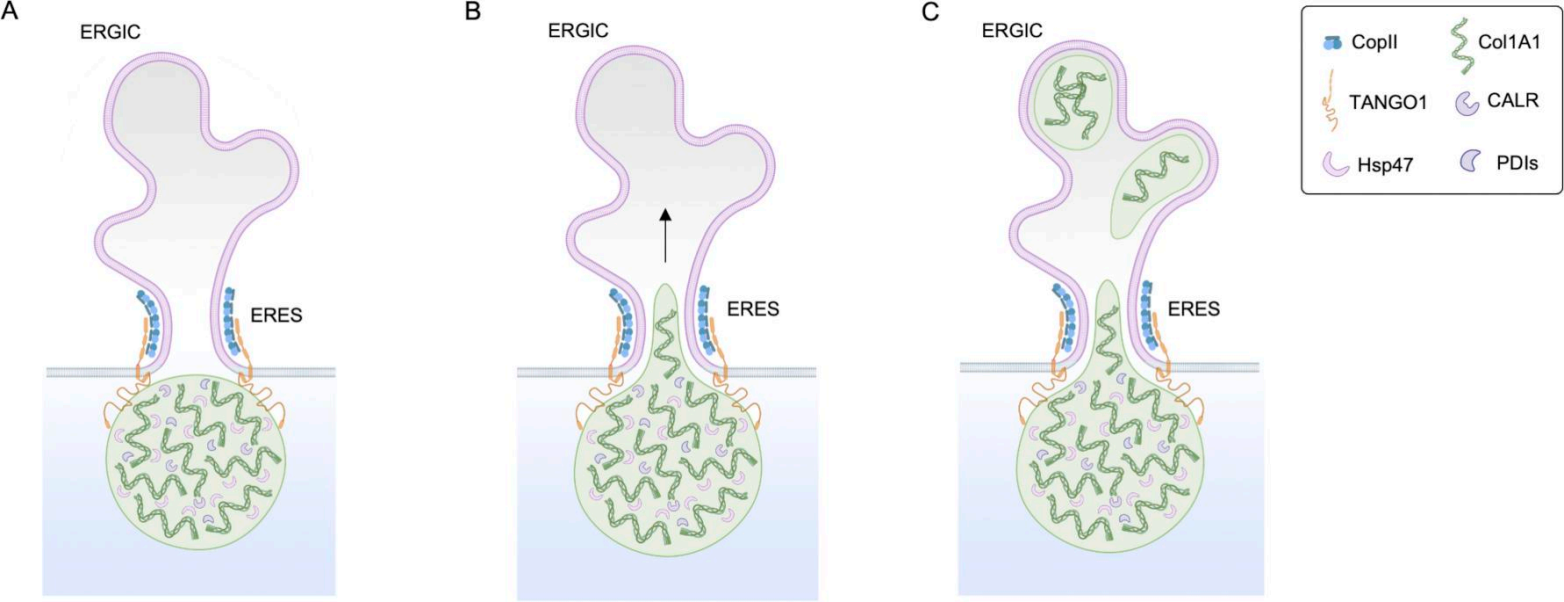
**Model for the liquid-like mode of PC1 export. (A)** ER luminal domain of TANGO1 captures PC1 condensates at ERES. **(B)** PC1 progresses from the ERES to the ERGIC due to capillary action or forced extrusion at the ER-ERES junction. **(C)** Changes in pH and/or calcium within the ERES-ERGIC conduit dissociate PC1 from chaperones. PC1 then moves forward along the secretory pathway, whereas TANGO1 is retained at the ERES. Elements in the figure have been generated using BioRender.

We propose that PC1 export proceeds through at least three coordinated stages (Fig. 5, A–C). First, condensates are captured at ERES through TANGO1-dependent interactions, consistent with the impaired secretion observed upon TANGO1 loss. Second, PC1 is extruded from the ER into the narrow export conduit, potentially driven by capillary forces or forced extrusion. Third, ionic changes, such as shifts in pH or calcium concentration, promote dissociation of PC1 from its chaperone network, enabling its release into the ERGIC, while TANGO1 remains at the ERES.

Within this framework, PC1 trafficking is better understood not as the vectorial transport of rigid supramolecular rods, but as the flow of a dynamic, deformable phase. Crucially, the challenge is not solely one of cargo size, but also of abundance and throughput—parameters whose quantitative contributions remain poorly defined.

The field has often favored mutually exclusive models of ER export, invoking either canonical COPII-coated carriers or TANGO1-mediated tunnels and tubules. However, the reported coexistence of both structures at ERES argues against such a binary interpretation (Nair et al., 2025; Weigel et al., 2021; Yang et al., 2021; Farhan et al., 2025). Rather than representing competing mechanisms, these pathways are likely deployed in a context-dependent manner, shaped by cargo properties, local concentration, cell type, and the physiological demands placed on the secretory system.

Accordingly, ER export may be more productively viewed as a continuum of intercompartmental cargo flow, in which distinct classes of cargo remain spatially segregated yet are mobilized through different transport architectures as required by cellular need.

## Materials and methods

### Cell culture

LX2 cells were obtained from Cytion (305039). LX2 cells were cultured in Dulbecco’s modified Eagle’s medium (DMEM; SH30243.01; Lonza) supplemented with 2% heat-inactivated fetal bovine serum (FBS; 10270-106; Gibco), 1× penicillin–streptomycin (15140122; Gibco), at 37°C in a humidified incubator supplied with 5% CO_2_. U2OS and HEK293T cells were cultured in DMEM supplemented with 10% FBS and 1× penicillin–streptomycin. All cells were regularly tested for *Mycoplasma* contamination. For all assays described in the results, the media were supplemented with 170 μM 2-phospho-L-ascorbic acid (49752; Sigma-Aldrich).

### Antibodies and reagents

The following primary antibodies were used: anti-Col1A1 (ab138492; Abcam), anti-α-SMA (ab124964; Abcam), anti-calnexin (ab22595, Abcam), anti-Hsp47 (ADI-SPA-470; Enzo), anti-GalNT (AF7507; Novus Biological), anti-calreticulin (ADI-SPA-601; Enzo), anti-GRP78/Bip (ab21685; Abcam), anti-LC3 (CAC-CTB-LC3-2-IC; Cosmo Bio), anti-G3BP1 (sc-365338; Santa Cruz), anti-P4HB/PDIA1 (ab2792; Abcam), anti-PDIA6 (18233-1-AP; Proteintech), anti-TANGO1 (HPA055922; Sigma-Aldrich), and anti-Sec31 (612350; BD Biosciences). The following secondary antibodies were used: donkey anti-mouse IgG-HRP (715035151; Jackson ImmunoResearch), donkey anti-rabbit IgG-HRP (715035152; Jackson ImmunoResearch), Alexa Fluor 488 donkey anti-rabbit (A21206; Invitrogen), Alexa Fluor 488 donkey anti-mouse (A21202; Invitrogen), Alexa Fluor 594 donkey anti-rabbit (A21207; Invitrogen), Alexa Fluor 594 donkey anti-mouse (A21203; Invitrogen), Alexa Fluor 647 donkey anti-mouse (A31571; Invitrogen), and Alexa Fluor 647 donkey anti-sheep (A21448; Invitrogen). The following reagents were used: recombinant human TGF-β1 (rcyc-htgfb1; InvivoGen), LysoTracker Red (L7528; Invitrogen), doxycycline hyclate (D9891; Sigma-Aldrich), thapsigargin (T9033; Sigma-Aldrich), polybrene (TR-1003; Sigma-Aldrich), Triton X-100 (T9284; Sigma-Aldrich), Tween-20 (P1379; Sigma-Aldrich), Hoechst-33342 (B2261; Sigma-Aldrich), Lipofectamine 3000 reagent (L3000015; Thermo Fisher Scientific), Lipofectamine RNAiMAX (13778150; Thermo Fisher Scientific), and protease inhibitor cocktail cOmplete (04693132001; Roche).

### Plasmids and siRNAs

We generated a mNeonGreen-tagged version of COL1A1 following an approach described previously (Lu et al., 2018). Briefly, we cloned the coding region of mNeonGreen between the COL1A1 signal sequence and the N-telopeptide, effectively replacing the COL1A1 N-propeptide. We PCR-amplified the COL1A1 signal sequence region (amino acids 1–24) from muscle cDNA, the mNeonGreen tag from an expression plasmid containing the mNeonGreen tag, and the COL1A1 coding region from the N-telopeptide until the C terminus from a plasmid encoding COL1A1 (amino acids 163–1,464). PCR fragments were subsequently used in a Gibson assembly reaction to assemble the ss-mNeonGreen-COL1A1 into a lentiviral expression plasmid, under the control of a doxycycline-responsive promoter, that was previously linearized by restriction enzyme digestion. The sequence of the final construct was verified by whole plasmid sequencing (Eurofins). For depleting TANGO1, two siRNAs (5′-GCA​AGA​AAC​UAG​UAU​GAU​UTT-3′, 5′-GCA​AUA​ACC​UCA​ACU​CUA​UTT-3′) targeting the ER luminal portion of TANGO1 were obtained from Eurofins. As controls, the following nontargeting siRNAs were used: 5′-UGG​UUU​ACA​UGU​CGA​CUA​ATT-3′, 5′-UGG​UUU​ACA​UGU​UGU​GUG​ATT-3′. Cells were transfected with 15 nM each of the siRNAs using Lipofectamine RNAiMAX following the manufacturer’s instructions.

### Collagen secretion assays

To measure amounts of type I collagen secreted by LX2 cells using western blot, 12 × 10^4^ cells were seeded in a 6-well plate. Cells were grown in the presence or absence of 10 ng/ml TGF-β for a total of 48 h. 24 h after the addition of TGF-β, media were replenished and these media were collected the next day. The collected media were spun at 800 g for 5 min to get rid of any detached cells and 20 μl of the supernatant media was resolved using SDS-PAGE and probed for Col1A1. For secretion assays using dot blots, TGF-β was added 24 h after seeding as before. After 48 h in TGF-β, the media were changed for Hanks’ balanced salt solution (HBSS) containing ascorbic acid and TGF-β and kept for 4 h after which it was collected, spun at 800 g for 5 min. 100 μl of the supernatant was allowed to pass through a 0.45-µm nitrocellulose blotting membrane (10600002; Amersham) assembled in a bio-blot microfiltration blotting system (1706545; Bio-Rad), after which the membrane was used to probe for Col1A1. For experiments involving RNAi of TANGO1, TGF-β was added 24 h after transfection with siRNAs and grown for another 48 h before changing the media for HBSS for 4 h and collecting the secreted fraction for dot blots. In all cases, the media were supplemented with freshly prepared 170 μM 2-phospho-L-ascorbic acid.

### Immunoblotting

Cells were lysed in buffer containing 1% Triton X-100, 500 mM NaCl, and 20 mM Hepes with 1× protease inhibitor cocktail. After lysis, samples were centrifuged at 10,000 *g* for 10 min to pellet the membrane fraction. The supernatant was collected, and total protein estimates were obtained using a Pierce BCA protein assay kit (23225; Thermo Fisher Scientific). Lysates containing an equal amount of total protein were resolved on an 8% polyacrylamide gel for SDS-PAGE. Proteins were transferred onto a 0.45-µm PVDF membrane (10600023; Amersham) for 3 h at a constant voltage of 80 V and blocked in 3% BSA in TBST. The membrane was incubated with primary antibodies overnight and washed 3 times for 5 min each with TBST before being incubated with secondary antibodies for 1 h. An enhanced chemiluminescence signal was generated by incubating the membranes with Immobilon Forte Western HRP Substrate (WBLUF0100; Millipore) and imaged on an iBright imaging system (Thermo Fisher Scientific). Membranes obtained from a dot blot assay were treated the same way with the exception of the protein transfer step.

### Generation of stable cell lines

0.8 × 10^6^ HEK293T cells were seeded in a 60-mm culture dish with 3 ml media. Lentiviral particles were generated by cotransfecting packaging plasmids for the second-generation lentivirus production system, pMD2G and pPAX2, along with a transfer plasmid carrying the Col1A1 sequence tagged to mNeonGreen under a doxycycline-inducible promoter using 6 μl TransIT-293 (Mirus Bio). The cell culture media were changed for fresh media the following day. 2 and 3 days after, the cell media were collected and filtered through a 0.45-µm membrane. LX2 cells were plated in a 60-mm culture dish, and the following day, the culture medium was changed for media containing the filtered viral particles diluted 1:1 in complete DMEM along with 4 µg/ml polybrene. The cells were grown in virus-containing media for 24 h after which fresh media were added. Col1A1-mNG expression was induced by the addition of 1 µg/ml of doxycycline overnight, and cells positive for mNeonGreen expression were sorted by fluorescence-activated cell sorting and expanded for subsequent assays.

### Immunofluorescence microscopy

Cells grown on glass coverslips were fixed with 4% paraformaldehyde in PBS for 12 min, washed, and permeabilized with 0.1% Triton X-100 for 6 min. Cells were blocked with 3% fatty acid–free BSA in PBS for 1 h and incubated with respective primary antibodies diluted in the blocking buffer overnight. After washes, cells were incubated with the respective secondary antibodies diluted in blocking buffer for 1 h. Cells were then washed and the coverslips mounted on glass slides with FluorSave (345789; Sigma-Aldrich). High-resolution fluorescence images were acquired in an inverted Leica STELLARIS confocal microscope with a 63× objective and suitable excitation lasers and equipped with photomultipliers and hybrid detectors. Unless otherwise mentioned, images represented in the results are maximum-intensity projections of z-stacks spanning the entire cell volume.

### FRAP and live-cell imaging

The FRAP experiments of PC1 condensates were done on an inverted Leica STELLARIS confocal microscope with a 63× objective. Cells were grown in 35-mm dishes with a polymer coverslip (81156; ibidi) and imaged at 37°C with 5% CO_2_. Two images were obtained prebleaching, bleaching was done with a 488-nm laser at 100% intensity, and images after bleaching were acquired at 2.5-s intervals. Live-cell imaging was done on an Olympus SpinSR spinning disk confocal system equipped with a temperature controller and CO_2_ chamber. Cells were grown in 35-mm dishes with a polymer coverslip (81156; ibidi) and imaged at 37°C with 5% CO_2_.

### Image analysis

Images were mainly processed using Fiji 1.54p (Schindelin et al., 2012). Colocalization analysis was performed using the JACoP plugin (Bolte and Cordelières, 2006) to obtain Manders’ colocalization coefficients. The FRAP recovery profiles were obtained on ImageJ and analyzed on GraphPad Prism and fit to an exponential one-phase association curve to obtain T_1/2._ For characterizing PC1 condensates, PC1 structures were segmented using the Spot Detector (Olivo-Marin, 2002) and Active Contours (Ulman et al., 2017) plugins implemented in Icy, version 2.5.4.0 (de Chaumont et al., 2012). Regions of interest (ROIs) corresponding to individual cells were manually delineated on microscopic images prior to automated analysis. Spot detection parameters were optimized to maximize the detection of PC1 structures and were set as follows: threshold scale 2 = 100 and threshold scale 3 = 80. The Active Contours plugin was subsequently applied to refine the PC1 ROIs detected by the Spot Detector. Parameters were adjusted to optimize segmentation accuracy: contour smoothness = 0.5, edge weight = −0.7, region weight = 1, contour sampling = 2, and maximum iterations = 1,000. Both plugins were executed within a single Icy protocol to automate and standardize the analysis workflow. For the histogram depicting PC1 condensate areas, only particles with a sphericity of 85 or above were considered and upper values were limited to two standard deviations from the mean. For quantitation of Col1A1 intensities in the ER and Golgi, ROIs corresponding to individual cells were manually delineated on confocal images using Icy. Confocal image stacks and cell ROIs were then imported into R. ROIs corresponding to the Golgi apparatus and ER were generated by applying the Otsu thresholding algorithm to sum-projected images of the GalNT and Hsp47 channels. Binary masks were converted into labeled ROIs using the bwlabel function (EBImage), which groups connected nonzero pixels into individual regions. ER and GA ROIs were spatially intersected with cell ROIs for each image, and the intensity of collagen I was quantified within these regions using sum-projected images of the corresponding channel. Data processing and analysis were performed in R using the following packages: ggplot2, dplyr, tidyverse, sf, terra, EBImage, stringr, and svglite. For the analysis involving Col1A1 condensate and Sec31 distances, the coupling index gives an estimation of the overlap between PC1 and Sec31 and is calculated by the SODA plugin as: sum {x in spots PC1, y in spots Sec31}(coupling probability (PC1, Sec31))/(total number of PC1).

### Statistical analysis

Statistical analysis was done using GraphPad Prism and R. Unless otherwise stated, all plots represent the mean with standard deviation of data points. The number of data points analyzed in each case and the associated *P* values have been stated in the figure legends. Statistical significance was calculated using two-tailed Student’s *t* test. For the coupling index and PC1-Sec31 distances, statistical significance of the measured differences was assessed using analysis of variance (ANOVA). The number of independent observations corresponded to the number of analyzed cells. For each cell, PC1-Sec31 separation distances were averaged prior to statistical analysis, and these per-cell mean values were used for ANOVA testing.

### Online supplemental material

Fig. S1 shows PC1 is intrinsically predisposed for LLPS. Fig. S2 shows PC1 condensates do not trigger a cellular stress response. Fig. S3 shows functional division of labor among ERES. Video 1 shows PC1 condensates undergo fission and fusion. Video 2 shows PC1 fluorescence within condensates recovers after photobleaching. Video 3 shows the liquid-like nature of PC1 in the ER. Video 4 shows PC1 condensates dissipate upon thapsigargin addition. Video 5 shows lysosomes do not colocalize with PC1 condensates.

## Supplementary Material

SourceData F1 is the source file for Fig. 1.

SourceData F4 is the source file for Fig. 4.

SourceData FS1 is the source file for Fig. S1.

## Data Availability

The data underlying all figures are available in the published article and its online supplemental material.
