# Supplementary figures and images for: Procollagen 1 assembles into phase-separated condensates in the endoplasmic reticulum
Source: J Cell Biol. 2026 Jun 11;225(8):e202603129. doi: 10.1083/jcb.202603129 (PMC13255678; doi:10.1083/jcb.202603129)

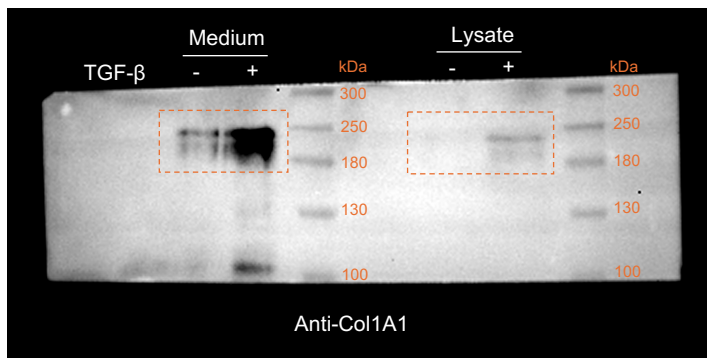

1.A.ii.

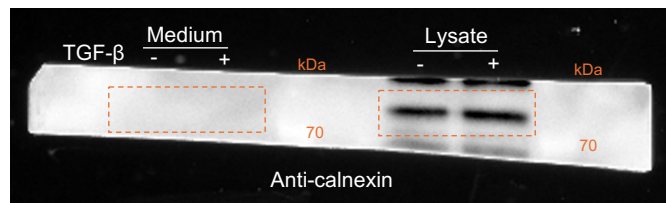

1.A.iii.

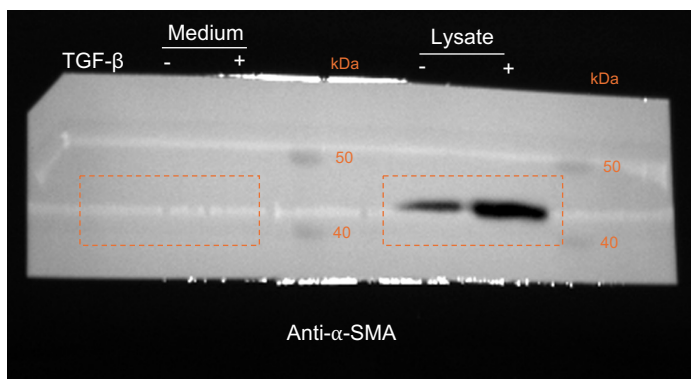

1.D.

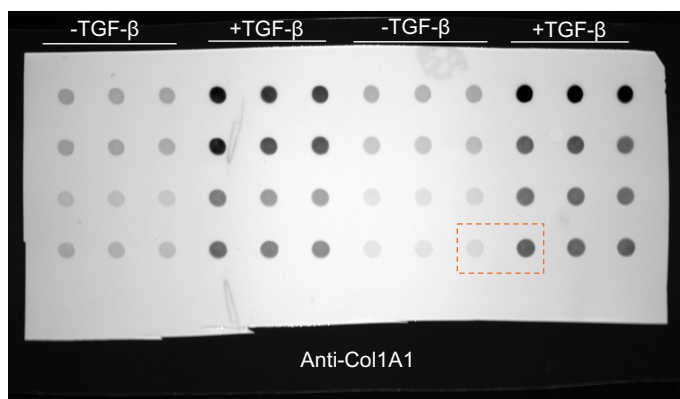

Supplement: SourceData F1 — is the source file for Fig. 1. [file jcb_202603129_sourcedataf1.pdf]

4.H.i.

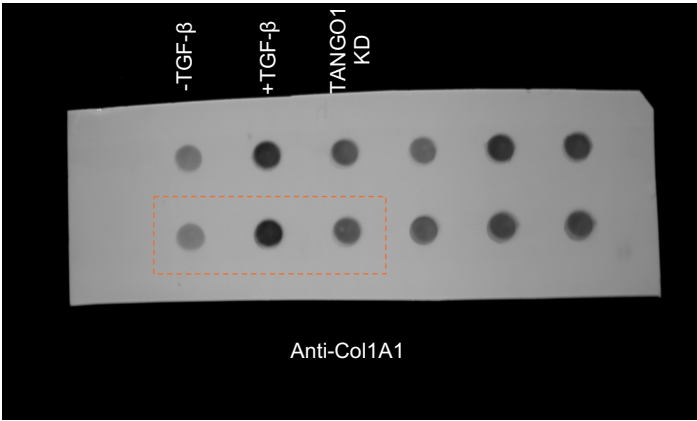

4.H.ii.

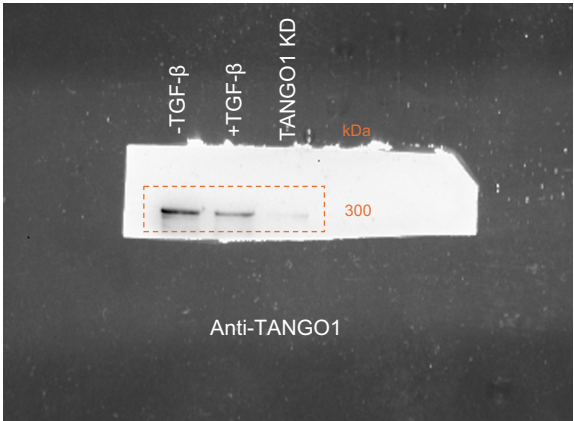

4.H.iii.

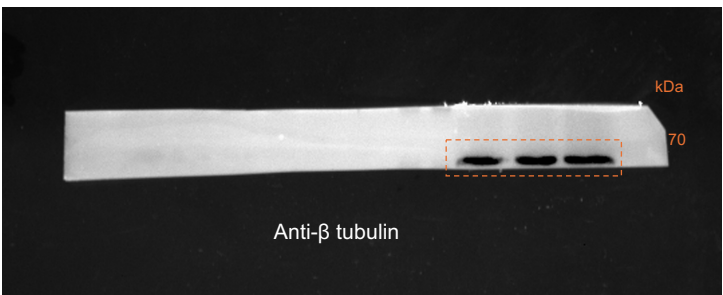

4.H.iv.

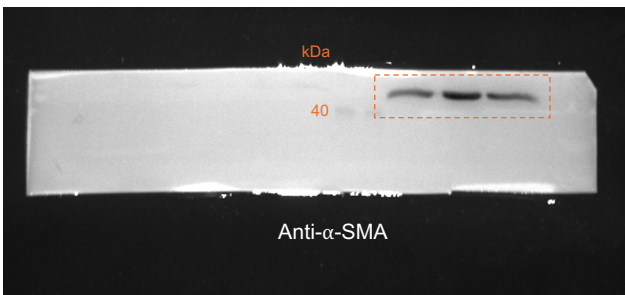

Supplement: SourceData F4 — is the source file for Fig. 4. [file jcb_202603129_sourcedataf4.pdf]

Supl.1.A.i.

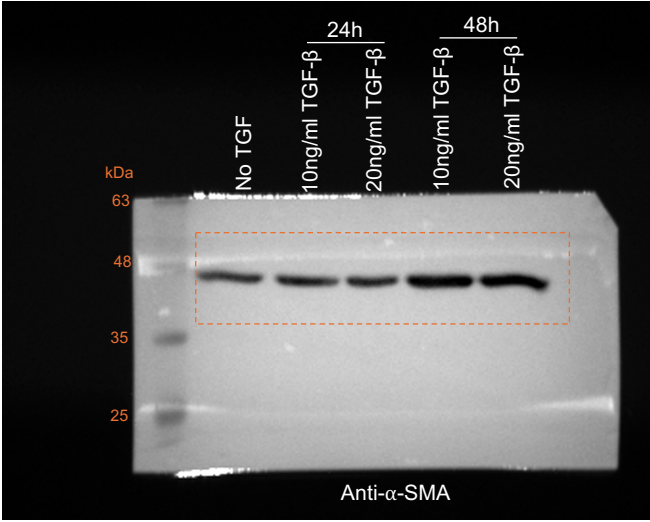

Supl.1.A.ii.

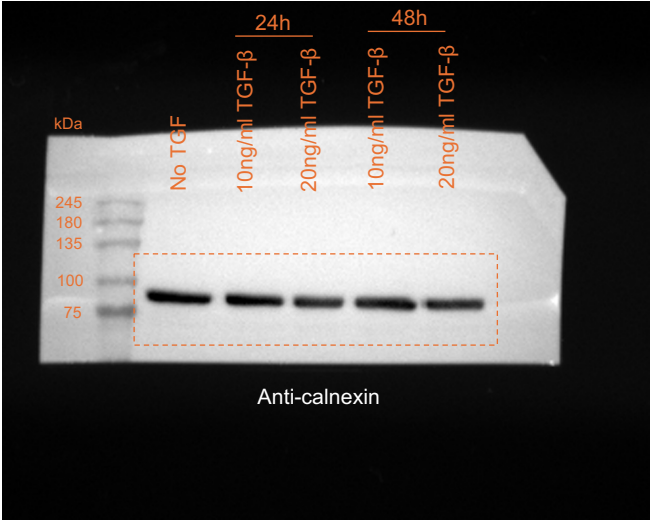

Supplement: SourceData FS1 — is the source file for Fig. S1. [file jcb_202603129_sourcedatafs1.pdf]
